# Introducing the Oxford Vocal (OxVoc) Sounds database: a validated set of non-acted affective sounds from human infants, adults, and domestic animals

**DOI:** 10.3389/fpsyg.2014.00562

**Published:** 2014-06-24

**Authors:** Christine E. Parsons, Katherine S. Young, Michelle G. Craske, Alan L. Stein, Morten L. Kringelbach

**Affiliations:** ^1^Section of Child and Adolescent Psychiatry, Department of Psychiatry, University of OxfordOxford, UK; ^2^Center of Functionally Integrative Neuroscience, Department of Clinical Medicine, Aarhus UniversityAarhus, Denmark; ^3^Department of Psychology, University of CaliforniaLos Angeles, CA, USA

**Keywords:** emotion perception, infant crying, distress calls, parent-infant, caregiving, auditory perception, infant vocalization

## Abstract

Sound moves us. Nowhere is this more apparent than in our responses to genuine emotional vocalizations, be they heartfelt distress cries or raucous laughter. Here, we present perceptual ratings and a description of a freely available, large database of natural affective vocal sounds from human infants, adults and domestic animals, the Oxford Vocal (OxVoc) Sounds database. This database consists of 173 non-verbal sounds expressing a range of happy, sad, and neutral emotional states. Ratings are presented for the sounds on a range of dimensions from a number of independent participant samples. Perceptions related to valence, including distress, vocalizer mood, and listener mood are presented in Study 1. Perceptions of the arousal of the sound, listener motivation to respond and valence (positive, negative) are presented in Study 2. Perceptions of the emotional content of the stimuli in both Study 1 and 2 were consistent with the predefined categories (e.g., laugh stimuli perceived as positive). While the adult vocalizations received more extreme valence ratings, rated motivation to respond to the sounds was highest for the infant sounds. The major advantages of this database are the inclusion of vocalizations from naturalistic situations, which represent genuine expressions of emotion, and the inclusion of vocalizations from animals and infants, providing comparison stimuli for use in cross-species and developmental studies. The associated website provides a detailed description of the physical properties of each sound stimulus along with cross-category descriptions.

## Introduction

Darwin (1872) was the first to describe and classify human emotional facial expressions and vocalizations as biologically determined expressions of inner emotions. Human facial expressions have since been the subject of a vast body of research, demonstrating that the basic categories of emotional facial expressions are highly recognizable and present across cultures (for review see Ekman, 1993). Although studied to a lesser extent, vocal expressions of emotion can also be readily categorized and occur across cultures (e.g., Scherer et al., 2001; Sauter et al., 2010a). Furthermore, both facial expressions and non-verbal emotional vocal expressions are ubiquitous across the life span, and apparent in infants from birth (Parsons et al., 2010, 2013a).

Facial expressions and vocal expressions clearly differ in the manner through which they convey emotion. In facial expressions, emotion is communicated via configurations of movements or positions of facial muscles (Ekman, 1993). In vocalizations, emotion can be communicated in two forms: verbal prosody and non-verbal affective bursts (Scherer, 1994). Verbal prosody provides information about the affective state of the speaker through the intonation and melody of speech. Non-verbal affective bursts, by contrast, have no linguistic content and tend to be more spontaneous, less constrained expressions of emotion, such as crying, screams, laughter, and sighing. These vocalizations typically accompany intense emotional states, and closely parallel affective vocalizations from other species (Scherer, 1995).

The majority of research into human emotional vocalizations to date has examined verbal prosody (for review see Scherer, 2003). Sound databases for this purpose generally contain vocalizations from actors speaking semantically neutral sentences or strings of “pseudo-words” with different types of prosody [e.g., the Danish Emotional Speech Database (Engberg and Hansen, 1996); Berlin Database of Emotional Speech, (Burkhardt et al., 2005)]. Controlling for the semantic content in this manner ensures that there is no interaction between meaning and prosody. However, such sentences have the disadvantage of being unnatural because there is typically a correspondence between semantic and prosodic features (Scherer et al., 1984). Another common method, using “pseudo-sentences” (sentences made up of non-words) again avoids semantic confounds, but is limited in terms of how natural the vocalizations sound. A second issue is that even pseudo-words often contain features that are language-specific, such as legal phoneme groups. In summary, the study of verbal prosody is, by necessity, entangled with features of the language being studied.

As a consequence, there has been increased interest in the use of non-verbal affective bursts as a means of studying emotion processing. These bursts constitute a primitive and universal mode of communication (Sauter et al., 2010a) and parallel the vocalizations of other species (e.g., Scherer, 1995; Juslin and Laukka, 2003; Belin et al., 2004). Some categories of vocal burst, such as laughs, cries and screams, are thought to reflect pure, “raw” emotion and therefore are powerful stimuli to use when investigating responses to emotion (Scherer, 1995).

Affective bursts are fundamentally different to speech in their underlying production mechanisms (Scott et al., 2010). Speech requires that specific physical parameters are produced, whereas affective bursts do not, allowing for the presence of a greater range of vocal features. The relative lack of language specific features means that sounds can be used to study universal or cross-cultural perception of emotion. Studies on cross-cultural perception of non-speech sounds are only beginning to emerge. Those that exist suggest that basic emotions can be recognized across diverse cultures (Sauter et al., 2010b; Koeda et al., 2013; Laukka et al., 2013) but perception of more elaborate emotional expressions, such as “triumph” (Sauter et al., 2010b), “disgust” (Koeda et al., 2013), or “shame” (Laukka et al., 2011) show cross-cultural differences. Like for faces, there may be universally recognized categories and also culture-specific signals (Elfenbein and Ambady, 2002).

While there are numerous well-established databases of faces and facial expressions, the study of vocal emotion processing has traditionally lagged behind. The available databases of affective bursts contain sounds generated by adult actors [e.g., The Montreal Affective Voices, (Belin et al., 2008); The International Affective Digitized Sounds, (Stevenson and James, 2008); Corpus of Non-verbal Vocalizations, (Lima et al., 2013)]. For instance, the Montreal Affective Voices database comprises vocalizations from ten actors, all of whom produced the vowel sound /a/ in discrete emotional categories. The Corpus of non-verbal vocalizations comprises eight emotional sound burst categories (four negative, four positive) from adults asked to imagine an emotion and make a corresponding vocal sound. A major advantage of these databases is that they include sounds with a range of emotions, which overall are highly recognizable (Belin et al., 2008; Lima et al., 2013).

However, the use of actors to obtain affective vocalization bursts is not without difficulty. Acted vocalizations are, by definition, not spontaneous emotional expressions. Reliance on acted vocalizations assumes that acted and authentic vocalizations are the same, or that differences in authenticity are imperceptible. However, there is growing evidence to suggest that authenticity in emotional vocalizations can be readily detected by adult listeners (Barker, 2013). Acted emotional expressions have been shown to be perceived as sounding more stereotyped and exaggerated (Laukka et al., 2011) and more extreme (Barkhuysen et al., 2007) compared to authentic ones. A number of physical differences between acted and authentic vocalizations have also been described, such as acted vocalizations having a higher and more variable pitch (Audibert et al., 2010; Jürgens et al., 2011). Finally, distinct patterns of neuronal activity have been found when listening to authentic compared to acted speech segments (Drolet et al., 2012), suggesting that differential processing occurs as a function of affective authenticity.

The reasons why authentic and acted vocalizations differ are not yet clear, but are likely to be variable and dependent on the quality of acting. It has been suggested that some fundamental features that support the identification of acted vocalizations (such as intensity of the emotion) may be unavoidable (Scherer, 2003). Other suggestions center around the idea that emotions are accompanied by physiological reactions (Kreibig, 2010) not under full voluntary control, that influence emotional expression in a way that is difficult to imitate (Scherer, 1986; Juslin and Laukka, 2001). Whatever the source of the differences, it is clear that future research on emotion processing would benefit from the availability of a database of authentic affective vocalizations.

Current understanding of auditory emotion processing has largely been derived from studying responses to adult emotional vocalizations. There are at least two other classes of emotional sounds that could inform our knowledge: vocalizations from younger humans and those from other species. For instance, it is hard to imagine sounds with greater biological salience than the vocal cues from infants. In the early postnatal period, vocalizations communicate degrees of distress, providing vital information for adults about an infant's needs (Soltis, 2004). Initially, the range of vocalizations an infant can produce is constrained by both the physiology of the developing vocal tract and the degree of muscular control over the vocal chords. As the infant develops, the range of vocal expressions becomes more sophisticated, incorporating more positive expressions such as laughter at around 4 months of age (Darwin, 1872; Sroufe and Wunsch, 1972; Nwokah et al., 1994). While adults respond to infant crying in the hope of terminating the sound, laughter promotes positive, rewarding social interactions between parent and infant. Around the same age, infants begin to “babble” (Oller and Eilers, 1988). Babbling comprises phoneme-like sounds, often repeated in meaningless strings, and is thought to be the precursor to language (Petitto and Marentette, 1991). When infants “babble,” they tend to be neither distressed nor excited, but in an emotionally neutral state (Rothgänger, 2003).

Vocalizations from animals, particularly of the domestic kind, share a number of similarities with infant vocalizations. They convey information about an animal's current needs and often serve to initiate caregiving responses. In particular, domestic cat meows and dog whines are familiar, easily recognizable sounds that tend to elicit sympathetic and caregiving responses from human hosts (Pongrácz et al., 2005). For instance, domestic cats can effectively use their characteristic vocalizations, purrs, to solicit human care (McComb et al., 2009). Despite some functional overlap between animal and infant vocalizations, these sounds are clearly distinctive and easily differentiated from human vocalizations.

Here, we present a database of authentic affective bursts from all three of these sound categories: human adults, human infants and domestic animals. The major aim of this database is to make available a set of multi-purpose, high quality, recognizable sounds for research across a broad range of domains. This new database has a number of advantages. First, the sounds are natural, from non-acted sources and therefore not affected by issues of authenticity. Second, in addition to adult vocalizations, this database includes a range of infant and domestic animal vocalizations. These sounds allow questions to be addressed such as, “What is special about human conspecific vocalizations?” and “How do infant vocalizations uniquely elicit parental care?” Third, categories of affective bursts considered to be especially effective in provoking emotion in the listener, such as laughs and cries (Scherer, 1995) along with less intense and emotionally neutral sounds, are included. These categories were included to allow comparison across a range of emotional vocalizations universally produced by infants and adults. In addition, these sound categories appear to be most readily recognized across cultures (e.g., Laukka et al., 2013). This paper describes the acquisition and standardization of this database of spontaneous, natural emotional vocalizations, referred to as the Oxford Vocal Sounds, “OxVoc” database (http://www.kringelbach.org/oxvoc).

## Methods

### Stimuli

#### Infant vocalizations

Infant vocalizations were obtained from video recordings of infants filmed in their own homes in the UK during a play and feeding session with their primary caregiver. This play and feeding session included periods of interactive play, a mealtime, and brief separation of the infant and caregiver. These video recordings were collected as part of a previous project with the approval of the Oxford Psychiatric Research Ethics Committee. In addition, permission for a validation study testing responses to these sounds was obtained from the Central University Research Ethics Committee. Recordings from nine male and female infants were used for the current database (five male).

Exemplars of different types of vocalizations were selected from video recordings. Cry vocalizations (*n* = 21) occurred primarily when infants were separated from their caregiver. Laughter vocalizations (*n* = 18) occurred primarily when the infant played with their caregiver. Neutral “babbles” (*n* = 25) were selected from periods of calm interaction between infant and caregiver, often during mealtime or play. All infants were full-term, healthy, and aged between 6 and 8 months at the time of recording (*M* = 6.7 months, *SD* = 0.9). Audio recordings were extracted from these videos and clips of vocalizations, free from background noise, were selected.

#### Adult vocalizations

Adult vocalizations were obtained from video diary blogs and video product reviews consisting of individuals speaking directly to a camera (primarily sourced through YouTube.com). The individuals included were all from the UK or the Unites States. Individuals who uploaded this content were contacted in order to obtain their permission for the anonymous use of short clips of vocalizations for research purposes. Video blogs were manually searched for instances of emotional and non-emotional vocalizations. For the distress vocalizations (*n* = 19), clips were taken from videos posted by females aged approximately 18–30 years. The cause of crying was variable, but often occurred when describing upsetting life events. No similar available videos of male adults crying were found. Laughter and neutral vocalizations were obtained from both males and females, aged approximately 18–30 years, occurring naturally during conversation. Thirty exemplars of laughter vocalizations (15 male and 15 female) and thirty exemplars of neutral vocalizations (15 male and 15 female) were collected.

#### Animal distress vocalizations

Animal distress vocalizations were obtained from freely available internet sources (e.g., www.freesound.org). Recordings of pet cats and dogs posted by their owners were manually searched, and relevant segments extracted.

### Physical matching methods

The duration of stimuli was standardized using Audacity software (version 1.3.4-Beta, http://audacity.sourceforge.net/). Matching of all other physical parameters was completed using Adobe® Audition® software (CS5 v4.0, Adobe System Corporation, San Jose, CA). All selected stimuli were free from background noise and were cropped to 1500 ms in length. The onset and offset amplitude envelope was standardized across all stimuli by applying linear rise and fall times of 150 ms to the start and end of each clip (“Volume Envelope” function). Stimuli were matched for total root-mean-square (RMS) amplitude for each clip to −25 dBFS (decibels full scale). This method matched the overall intensity of the clip, while retaining variations in amplitude envelope across time.

Basic physical properties of vocalization stimuli including fundamental frequency, duration of vocal “bursts” within each stimulus and number of vocal bursts per stimulus, are presented in Table 1. Full details of physical parameters for each individual stimulus are included in the Supporting Information.

**Table 1:** **Basic physical parameters of vocalization stimuli within the OxVoc database**.

**Stimulus category**	**Number of stimuli**	***F*_0_ (Hz), *M* (*SD*)**	**Number of vocal bursts, *M* (*SD*)**	**Mean burst duration (s), *M* (*SD*)**
Infant cry	21	445.54 (84.81)	1.90 (1.09)	1.02 (0.50)
Infant neutral	25	347.34 (122.34)	1.88 (0.97)	0.93 (0.46)
Infant laugh	18	348.31 (87.95)	3.22 (1.40)	0.41 (0.22)
Adult cry	19	368.22 (94.83)	2.11 (0.32)	0.55 (0.130)
Adult neutral	30	228.13 (57.88)	1.00 (0.00)	0.91 (0.20)
Adult laugh	30	348.83 (104.12)	4.27 (1.78)	0.37 (0.29)
Animal distress	30	439.28 (101.53)	1.63 (0.76)	1.01 (0.42)
Total	173			

Values presented are averaged across stimuli within each stimulus category individual category.

### Ratings of stimuli

A number of independent samples of participants were asked to listen to and rate the presented sounds on multiple dimensions. First, three independent samples of participants rated a large set of stimuli on three dimensions related to the emotional experience of the sounds (Study 1). Participants rated the distress of each sound, the mood of the individual producing the sound and also their own emotional reaction to the sound. Second, a further independent sample of participants rated the sounds on three additional dimensions: arousal of the sound producer, the valence of the sound, and the perceiver's motivation to respond to the sound (Study 2). The valence and arousal scales were selected to allow direct comparisons between ratings of the current stimuli and those from acted databases (Belin et al., 2008; Koeda et al., 2013; Lima et al., 2013). This latter “motivation to respond” rating was obtained because experiencing an emotional cue can be divided into its subjective appraisal (“liking”) and also its incentive salience (“wanting”; Berridge and Kringelbach, 2008). This is of relevance to how the present sounds are perceived because previous work has demonstrated gender differences in “wanting” and “liking” of infant cues (Parsons et al., 2011).

### Participants

Participants were recruited from the student and general UK population via posters and online advertisement. Inclusion criteria for participation were: currently resident in the UK, normal hearing, not currently taking medication affecting the brain and no self-reported mental health problems.

### Study 1: three dimensions of emotional experience of sound

#### Infant vocalizations

Sixty-one participants (30 male; aged *M* = 27.58 years, *SD* = 6.87) rated 64 infant vocalization stimuli (21 cries, 18 laughs, 25 babbles). For each stimulus, participants rated “How distressed do you think the baby was?” on a 7-point Likert scale, with 1 as “not distressed” and 7 as “very distressed.” Participants also responded to “Please rate the mood of the baby” and “How did you find the sound?” on 9-point Likert scales, with −4 as “very negative” and +4 as “very positive.” Participants listened to each stimulus once, after which they completed all three ratings before moving on to the next stimulus. The order of stimuli was pseudo-randomized across participants. Stimuli were presented using WinAmp software (Winamp v 5.61 Nullsoft, Inc., http://www.winamp.com), through Sony in-ear earphones (MDR-EX77LP). Responses were made by circling the appropriate value on the Likert scale using pen and paper. This procedure took approximately 15 min in total. For ease of comparison, data were converted to standardized scores (*z*-scores).

#### Adult and animal distress vocalizations

An independent sample of 24 participants (eight male; age *M* = 27.67 years, *SD* = 11.72) rated 49 adult and animal distress vocalizations. This set of stimuli consisted of female adult distress cries (“sobs” as opposed to screams, *n* = 19) and domestic animal distress vocalizations (cat meows, *n* = 15; dog whines, *n* = 15). A similar set of ratings to that described above for the infant vocalizations was collected. Participants responded to: (i) “how distressed do you think the adult/animal was?” from “very distressed” to “not distressed”; (ii) “please rate the mood of the animal,” from “very negative” to “very positive”; and (iii) “how did you find the sound?” from “very negative” to “very positive.”

**Figure 1:**
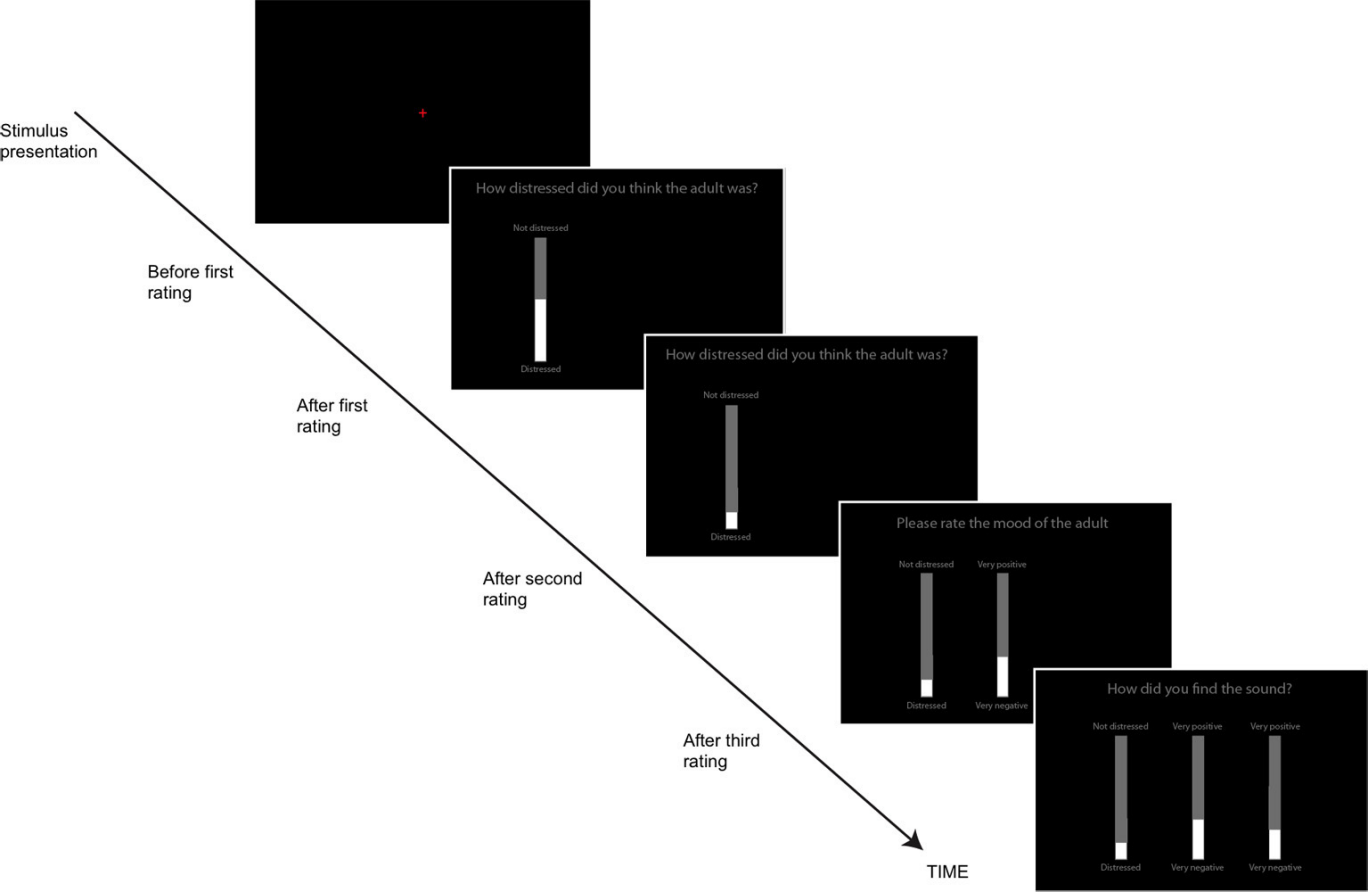
**The Visual Analog Scales used to rate the sounds on different dimensions (adult and animal sounds Study 1, all sounds, Study 2)**. Participants first heard the sound (red fixation cross on screen), then made three ratings sequentially.

Participants responded using computerized visual analog scales (VAS), implemented as vertical onscreen bars. A gray bar indicated the total range of possible responses, while a slightly narrower white bar, centered on top of the gray bar indicated the participants' current response. The height of the white bar could be altered by using the “UP” and “DOWN” arrows on the keyboard. After each stimulus was presented, individual scales appeared on the screen along with the question to be rated (see Figure 1). This task was self-paced; participants moved from one rating to the next by pressing the spacebar and the task lasted approximately 12 min in total. Participants' responses were again converted to standardized scores.

#### Other adult vocalizations: happy and neutral

An additional sample of 25 participants (seven male; age *M* = 29.12 years, *SD* = 9.7) rated 60 adult vocalizations. This set of stimuli consisted of equal numbers of male and female adult neutral vocalizations (15 each) and equal numbers of males and female happy (laugh) vocalizations (15 each). Participants again responded to: (i) “how distressed do you think the adult was?” from “very distressed” to “not distressed”; (ii) “please rate the mood of the adult,” from “very negative” to “very positive”; and (iii) “how did you find the sound?” from “very negative” to “very positive.” The ratings procedure was identical to that described for the adult and animal distress vocalizations.

## Results

Across all participant ratings, reliability was found to be very high, with intraclass correlations (ICC) > 0.90. Therefore, data is combined across participants throughout (similar to the procedure reported in Tottenham et al., 2009). The relationship between ratings on the three dimensions (distress, stimulus mood, participant mood) across all stimuli was assessed using Pearson correlations. Clear correlations in ratings emerged across all three rating dimensions. There was a strong positive correlation between perceived distress and perceived stimulus mood [*r*_(173)_ = 0.92, *p* < 0.001], a strong positive correlation between perceived distress and participant mood [*r*_(173)_ = 0.94, *p* < 0.001] and a strong positive correlation between stimulus mood and participant mood [*r*_(173)_ = 0.97, *p* < 0.001].

Given the high correlations between all ratings, a factor analysis was performed to investigate whether these measures loaded onto a single underlying variable. The Kaiser-Meyer-Olkin (KMO) measure of sampling adequacy was 0.73 and Bartlett's test of sphericity was significant [χ^2^_(3)_ = 858.64, *p* < 0.001], justifying dimension reduction. A principal components analysis revealed the first factor explained 96.15% of the variance across ratings. This component is referred to as “valence.”

Differences in “valence” across types of emotional vocalization were then assessed using a One-Way ANOVA, using stimulus type (positive, negative, or neutral) as a factor. This analysis demonstrated a significant main effect of stimulus type on perceived valence [*F*_(2, 172)_ = 418.25, *p* < 0.001, *r* = 0.84]. *Post-hoc* Bonferroni comparisons demonstrated significant differences in perceived valence in pairwise comparisons of positive (infant and adult laughter), negative (infant, adult, and animal distress vocalizations), and neutral (infant and adult babbles) stimuli (all *p* < 0.001). Comparisons of valence between individual stimulus categories were carried out in Study 2 where all stimuli were rated by a single group of participants.

### Study 2: perceptions of arousal, valence, and motivation to respond to the sounds

#### Participants

Thirty-four participants (18 male) aged between 18 and 22 years (*M* = 20, *SD* = 0.9) took part in Study 2. Participants were recruited from a UK university population using online advertisements. All participants reported having normal hearing, were not parents, were not currently taking medication affecting the brain and had no self-reported mental health problems.

#### Method

Participants listened to all 173 stimuli and made three ratings for each sound using computerized Visual Analog Scales. Sounds were presented in a random order. The three ratings obtained were: “arousal,” “motivation to respond,” and “valence.” For the arousal measure, participants were asked, “Please rate how excited or calm you thought the sound was,” from, “very excited” to “very calm” (consistent with Bradley and Lang, 2007). For the motivation to respond measure, participants were asked “How much do you want to respond to the sound?” from “very much” to “not at all.” For the valence measure, participants were asked, “Please rate how happy or sad you thought the sound was,” from “very happy” to “very sad.” As for Study 1, participants listened to each stimulus once, after which they completed all three ratings before moving on to the next stimulus.

#### Results

Ratings of motivation to respond to the sounds, arousal, and valence of the sounds were examined using One-Way repeated measures ANOVAs. Differences between the specific stimulus categories were examined using paired samples *t*-tests with Bonferroni adjusted alpha levels of 0.007 (0.05/7).

#### Motivation to respond

Ratings of motivation to respond to the sounds differed significantly across the seven categories [*F*_(6, 192)_ = 39.12, *p* < 0.0001, *r* = 0.41]. Overall, infant vocalizations (cries, laughs, and babbles) elicited higher motivation to respond (*M* = 1.15, *SD* = 0.54) than the equivalent adult sounds [*M* = 0.26, *SD* = 0.68; *t*_(33)_ = 6.22, *p* < 0.0001, *r* = 0.73]. The adult neutral vocalizations stood apart from the other stimulus categories, receiving the lowest ratings of motivation to respond of all (all *p* < 0.0001).

**Figure 2:**
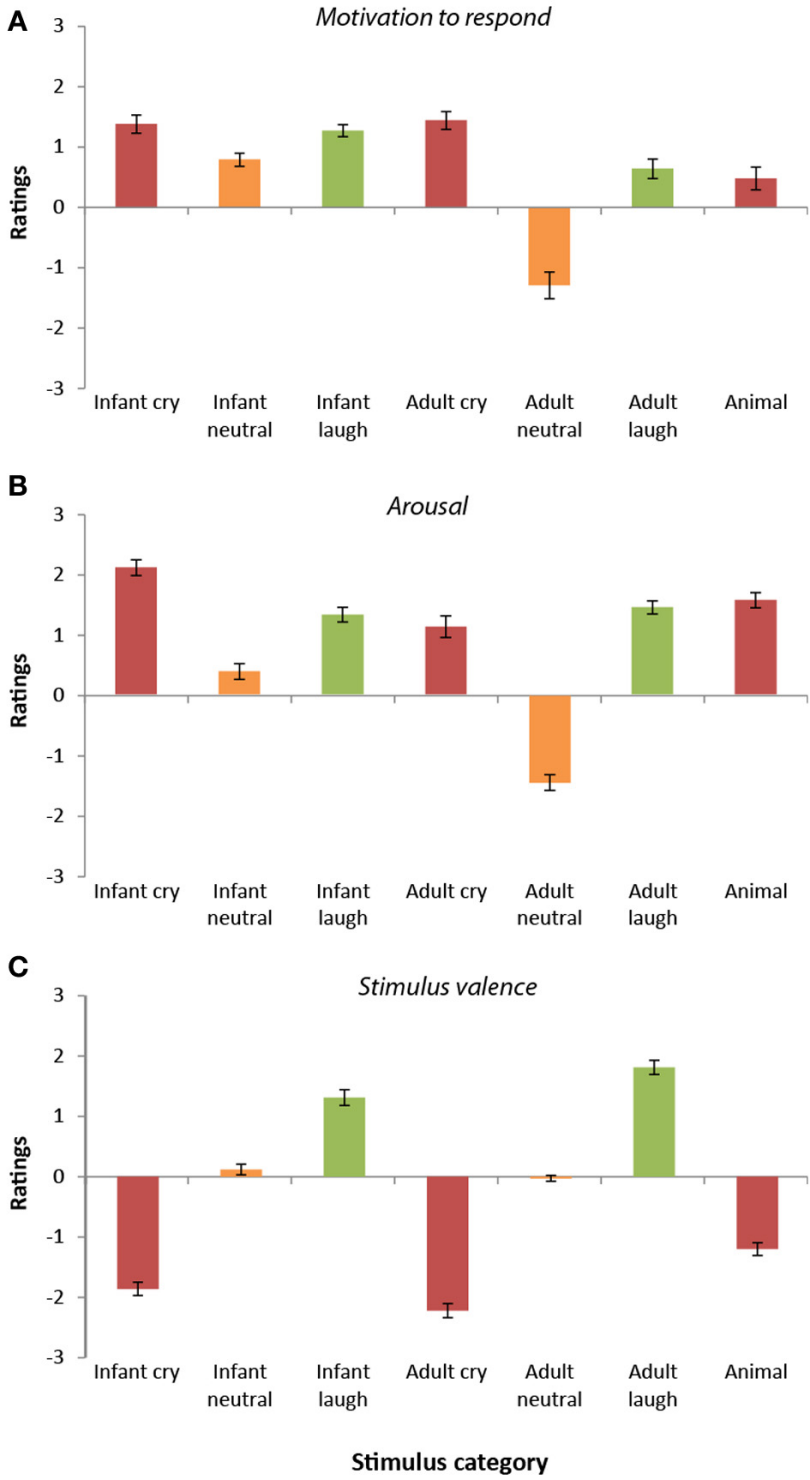
**Participant ratings of dimensions (Study 2). (A)** Ratings of motivation to respond varied across stimulus categories. Ratings were highest for cry vocalizations (both infant and adult) and lowest for adult neutral vocalizations. In general, motivation to respond to infant vocalizations was greater than that for adults. **(B)** Ratings of arousal were higher for emotional vocalizations (cries and laughter) compared with neutral vocalizations. Arousal was generally rated as higher in infant vocalizations compared with adult vocalizations. **(C)** Ratings of valence differed across categories. Valence ratings were lowest for cry/distress vocalizations and highest for laughter vocalizations.

Participant ratings of motivation to respond were highest overall for the infant cries, the adult cries, and the infant laughter (see Figure 2A). These three sound categories did not differ significantly from one another (all *p* > 0.55). Participants' ratings of motivation to respond to infant crying were significantly higher than for the infant neutral sounds [*t*_(33)_ = 4.09, *p* < 0.0001, *r* = 0.58], the adult neutral sounds, [*t*_(33)_ = 8.43, *p* < 0.0001, *r* = 0.83], the adult laugh sounds [*t*_(33)_ = 2.79, *p* < 0.009, *r* = 0.44], and the animal sounds [*t*_(33)_ = 8.48, *p* < 0.0001, *r* = 0.83].

The adult cry sounds were also given higher “motivation to respond” ratings in comparison to the two other categories of adult vocalization [neutral, *t*_(33)_ = 9.34, *p* < 0.0001, *r* = 0.85; laughter, *t*_(33)_ = 3.64, *p* < 0.0001, *r* = 0.54], the animal sounds [*t*_(33)_ = 5.23, *p* < 0.0001, *r* = 0.67] and the infant neutral sounds [*t*_(33)_ = 3.99; *p* < 0.0001, *r* = 0.57]. Participants' rated motivation to respond to infant laughter was significantly higher than for the infant neutral sounds [*t*_(33)_ = 5.39, *p* < 0.0001, *r* = 0.68], the adult neutral sounds, [*t*_(33)_ = 8.43, *p* < 0.0001, *r* = 0.83], the adult laugh sounds [*t*_(33)_ = 2.79, *p* < 0.009, *r* = 0.44], and the animal sounds [*t*_(33)_ = 8.48, *p* < 0.0001, *r* = 0.83]. Motivation to respond was rated similarly for the other stimulus categories (*p* > 0.05).

#### Arousal

Ratings of arousal within the sounds also differed significantly across the seven categories [*F*_(6, 192)_ = 114.93, *p* < 0.0001, *r* = 0.61, see Figure 2B]. Like the “motivation to respond” ratings, infant vocalizations (cries, laughs, and babbles) were rated as having higher arousal levels (*M* = 1.27, *SD* = 0.62) than the equivalent adult sounds [*M* = 0.37, *SD* = 0.54; *t*_(33)_ = 9.54, *p* < 0.0001, *r* = 0.86]. Again, in keeping with the “motivation to respond” ratings, arousal ratings were lowest in response to the adult neutral sounds and highest for the infant crying (all comparisons, *p* < 0.0001). Adult crying was rated as higher in arousal than the infant neutral and adult neutral vocalizations (*p* < 0.0001), but was rated similarly to the other vocalization subtypes (all *p* > 0.08). Infant neutral sounds were rated as having higher arousal levels than the adult neutral sounds [*t*_(33)_ = 14.98, *p* < 0.0001, *r* = 0.93], but significantly lower levels of arousal than all other stimulus categories (all *p* < 0.0001). Infant laughter, adult laughter and animal sounds were all rated similarly in terms of their arousal levels (*p* > 0.05).

#### Valence

Ratings of the valence of the sounds differed significantly across the seven categories [*F*_(6, 192)_ = 200.00, *p* < 0.0001, *r* = 0.71; see Figure 2C]. Adult cries received the most negative ratings of valence (all comparisons *p* < 0.0001), followed by infant cries (again, all comparisons, *p* < 0.0001). In contrast, adult laughter received the most positive ratings of valence (all comparisons, *p* < 0.0001), followed by infant laughter (all comparisons, *p* < 0.0001). Infant neutral vocalizations and adult neutral vocalizations received similar valence ratings [*t*_(33)_ = 1.35, *p* = 0.86, *r* = 0.23], and were both rated as significantly less negative than the animal distress vocalizations [infant neutral vs. animal vocalizations; *t*_(33)_ = 9.7, *p* < 0.0001, *r* = 0.86; adult neutral vs. animal vocalizations; *t*_(33)_ = 8.68, *p* < 0.0001, *r* = 0.83].

#### Gender differences

Independent samples *t*-tests showed that there were no gender differences in ratings of motivation to respond to the sounds [*t*_(32)_ = 0.71, *p* = 0.48, *r* = 0.12], arousal levels of the sounds [*t*_(32)_ = 0.96, *p* = 0.38, *r* = 0.17], or in valence of the sounds [*t*_(32)_ = 1.83, *p* = 0.08, *r* = 0.31].

#### Relationship between ratings of motivation to respond, arousal, and valence

Examining all 173 sound stimuli, there was a significant relationship between participants' ratings of their motivation to respond to the sound and ratings of the arousal of the sound (*r* = 0.796, *p* < 0.0001; two tailed). There was no significant relationship between valence and motivation to respond (*r* = −0.12, *p* = 0.09) or between valence and arousal level (*r* = −0.13, *p* = 0.11).

Sounds were then divided into three categories, “infant,” “adult,” and “animal” to examine whether the relationships between the three different scales (motivation, arousal, valence) varied across categories. For the infant sounds, there was a strong positive correlation between ratings of arousal and motivation to respond (*r* = 0.59, *p* < 0.0001) and a strong negative correlation between arousal and valence (*r* = −0.42, *p* < 0.0001). However, there was no correlation between motivation to respond and valence (*r* = −0.15, *p* = 0.25). For the adult sounds, again there was a very strong positive correlation between ratings of arousal and motivation to respond (*r* = −0.90, *p* < 0.0001). In contrast to the infant vocalizations, there was no significant correlation between ratings of arousal and valence (*r* = −0.19, *p* = 0.09). Finally, as for the infant sounds, there was no significant correlation between ratings of motivation to respond and valence (*r* = −0.15, *p* = 0.19). For the animal sounds, as for the infant and adult sounds, there was a positive correlation between ratings of arousal and motivation to respond (*r* = −0.43, *p* < 0.01). There was also a negative correlation between ratings of arousal and valence (*r* = −0.43, *p* < 0.01), similar to the infant sounds. Finally, consistent with both the infant and adult sounds, there was no correlation between ratings of motivation to respond and valence (*r* = −0.35, *p* = 0.06).

## Discussion

The Oxford Vocal (“OxVoc”) Sounds database consists of 173 natural, spontaneous non-verbal affective vocalizations from infants, adults, and domestic animals. For the human sounds, the vocalizations are further subdivided into three discrete emotional categories, positive “laughs,” neutral “babbles,” and negative, “cries.” For the animal sounds, familiar distress sounds from two of our most common domestic companions, cats and dogs, are included. A detailed analysis is presented of how adults perceive these sounds on dimensions of distress, mood and listener mood (Study 1) as well as valence, arousal, and how motivated they are to respond to them (Study 2).

Adults rated the emotional content (“valence”) of the stimuli in both Study 1 and Study 2 consistent with the experimenter defined categories. For example, while sounds categorized as cries were rated as negatively valenced, laughter was rated as positively valenced. In Study 2, some striking differences emerged across the adult, infant and animal categories. For measures of “valence,” the adult stimuli received the most extreme ratings overall. Adult cries were rated more negatively than infant cries, and adult laughter was rated more positively than infant laughter. Both categories of human distress sound were rated more negatively than animal distress sounds. Neutral sounds from infants and adults were indistinguishable in terms of their rated valence.

The cause of the difference in ratings of valence in adult and infant vocalizations was not investigated in this study. It might be hypothesized that these differences are related to individual's attributions regarding the cause of vocalizations. For example, the cause of infant crying is often related to readily controllable environmental factors, such as hunger or separation from a caregiver. The cause of adult crying on the other hand, is often related to less controllable factors, such as loss or failure. It might be anticipated that, in line with attribution theory (Weiner, 1985), the greater “controllability” of infant crying compared with adult crying would impact the appraisal of these sounds. Indeed, previous research has demonstrated that even within infant vocalizations, individuals' perceived efficacy in caregiving, and presumably their ability to terminate infant distress, was related to how negatively infant cries were rated (e.g., Verhage et al., 2013). Despite equivalence in rated valence, adults were significantly more motivated to respond to the infant neutral vocalizations compared to adult neutral vocalizations. Furthermore, while adult positive and negative vocalizations were rated more extremely than the equivalent infant vocalizations, “motivation to respond” was greater overall for the infant sounds. These ratings suggest that sounds from an infant, be they distressed, neutral or positive, can especially motivate a response from an adult listener. This is consistent with a body of literature showing increased autonomic arousal and superior motor performance after listening to infant cries compared with other sounds, including adult cry sounds (Boukydis and Burgess, 1982; Zeskind and Collins, 1987; Bakermans-Kranenburg et al., 2012; Parsons et al., 2012). In addition, the potential for greater “controllability” of an infant's affective state, relative to that of an adult, may at least partly explain greater motivation to respond to these sounds.

Crying, from infants and adults alike, evoked the strongest ratings of motivation to respond. This is consistent with models of human empathy (e.g., Decety et al., 2012), which posit specific sensitivity to distress signals from others. While crying is relatively rare in adulthood (Zeifman, 2001), and often occurs when alone (Frey, 1985), it can serve to induce sympathy, empathy and comforting behavior and can help strengthen bonds between people (Vingerhoets et al., 2000).

The sound categories (infant, adult, and animal) also differed significantly with regards to their rated arousal. As for “motivation to respond” ratings, infant sounds were rated as having higher arousal levels than adult sounds. The relationship between emotion subcategory (e.g., cry) and rated arousal also differed dependent on who produced the sound (adult, infant). Generally the negatively valenced sounds were rated as highest in arousal, followed by the positively valenced sounds and then the neutral sounds. A comparable non-linear relationship between valence and arousal level in sound has been described previously (Bradley and Lang, 2007).

The lack of a linear relationship between ratings of arousal and valence was also evident in the correlational analysis from Study 2. The most robust finding from this analysis, occurring overall and for infant, adult and animal categories, was a positive correlation between arousal and motivation to respond. Sounds rated as “very excited” tended to elicit high “motivation to respond” ratings. For the infant and animal sounds, there was also a correlation between ratings of arousal and valence. However this relationship was not present in ratings of the adult sounds, and did not emerge in analyses of the sounds overall. These findings indicate that there are considerable differences in how adults perceive arousal and valence in similar vocalizations from adults, infants and animals.

We did not find any significant differences in men and women's ratings of the OxVoc sounds. While there is evidence that women show advantages in certain aspects of auditory emotion processing (e.g., emotion identification; for review, see Schirmer and Kotz, 2006), these effects are small in nature (e.g., Bänziger et al., 2012) and certainly not apparent across the board. Women have been shown to be more accurate at emotion recognition (Belin et al., 2008) and some differences in cortical processing of speech prosody have been reported (Schirmer et al., 2004, 2005). However, a body of evidence has shown no differences between men and women in ratings of non-verbal vocalizations (e.g., Hawk et al., 2009; Lima et al., 2013; Sauter et al., 2013), in response to speech prosody (e.g., Paulmann et al., 2008) or even in ratings of infant emotional cues (Parsons et al., 2011). Taken together, these findings suggest that any gender differences in response to emotional vocalizations, if they exist, are small in magnitude.

The OxVoc represents the first database of its kind to compile non-acted affective vocalizations from adults, infants and animals. The database includes human stimuli of variable valence, from distress cries, to laughter, to more neutral “babble” sounds. We have demonstrated significant differences in adults' motivation to respond to these sound categories, and also in perception of arousal and valence within the sounds. As well as a detailed analysis of overall responses to the sound categories, we provide information on adults' responses to each individual sound stimulus and details of the physical properties of the sounds (Supplementary information). OxVoc will be made available, with appropriate ethical approval, for non-commercial research at established academic institutions. Interested researchers can refer to the OxVoc web page (add link here) for detailed information about accessing the material.

There are, of course a number of limitations to the OxVoc database. While an effort was made to include stimuli from a variety of important and underexplored emotional categories (e.g., infant and adult vocalizations, domestic animal calls), the selection process was necessarily one of convenience. We cannot make any claims as to how representative our set of vocalizations is of the entire range that exists. In obtaining multiple ratings on the same sounds, we did not counterbalance the order of rated dimensions. This was done to ensure simplicity for participants, but could be changed in future studies.

Useful future additions to the current database, or indeed any future database, might include: (i) a set of adult male cry vocalizations; (ii) vocalizations from children, adolescents and adults of different ages, (iii) vocalizations from individuals with different language backgrounds. Ratings reported here were from UK residents, so replication in another country and culture would be of considerable interest. Ultimately, it is our intention to add these stimulus categories and ratings in the future and describe both as we have done for the current set of sounds.

In conclusion, there are several strengths to the OxVoc, rendering it a unique and versatile corpus that can be used for many different research purposes. First, these sounds were obtained from non-acted sources, and are as close to spontaneous, genuine expressions of emotion as possible. Second, the inclusion of vocal expressions from animals and infants provides comparison stimuli for use in cross-species and developmental studies. For instance, comparisons of responses to human and non-human vocalizations would allow the investigation of hypotheses regarding selective tuning to conspecific emotion. Third, ratings are available for these stimuli on a range of dimensions. The availability of such information means that the stimuli can be used for many different purposes, dependent on experimenter requirements. For instance, the sounds have already been used to examine the neural processing of infant cues (Parsons et al., 2013a,b), detection of functionally important physical characteristics of vocalizations (Young et al., 2012) and movement in response to sound salience (Parsons et al., 2012). Other possibilities include investigation of physiological responses to the sound categories (e.g., infant sounds) or to the specific dimensions (e.g., high arousal sounds) independent of category.

Fourth, the variability of adults' motivation to respond to these sounds provides intriguing opportunities to investigate the relationship between emotion perception, action and attributes of the vocalizer. Fifth, the associated website (http://www.kringelbach.org/oxvoc) provides a detailed description of the physical properties of each sound stimulus, along with cross-category descriptions.

### Conflict of interest statement

The authors declare that the research was conducted in the absence of any commercial or financial relationships that could be construed as a potential conflict of interest.
